# The New Clinic at Liverpool Royal Infirmary

**Published:** 1918-07-13

**Authors:** 


### The New Clinic at Liverpool Royal Infirmary.

The recently established electro-therapeutic and orthopædic out-patient department at the Liverpool Royal Infirmary has been formally opened by Col. Sir Robert Jones. The soldier is still treated at Alder Hey Hospital, the parent institution. The new clinic at the Royal Infirmary is intended for the army pensioner in civil life, who will find it more accessible. The suggestion came from the War Pensions Committee, and the cost has been borne by the committee of the Royal Infirmary, which has installed the new department in the recreation hall. In addition to discharged men civilians will, of course, be eligible for treatment. Sir Robert Jones, on the opening day, emphasised the debt due to Professor Rushton Parker, who was the first to introduce the Thomas splint to any institution in Liverpool. If the need arises, an evening clinic will be established for the convenience of men who are at work during the day.

The main room, which was the concert hall, is about 100 ft. by 28 ft., with a raised platform for the gymnastic apparatus. The large "green room," with sanitary wings behind, is now the "baths" room and dressing-rooms. There are two rooms leading to the department, but divided from it by a large lobby, which makes an excellent waiting-room. The first is a surgeon's examination-room fitted with testing apparatus, and the latter contains the diathermy and high-frequency apparatus. The teaching massage sister is in charge with an assistant, and when the full number of patients arrive there will be twelve certificated masseuses (I.S.T.M. and A.P.M.M.C.) in addition. These will be exclusive of the pupils attached to the Royal Infirmary's Massage School, which Miss Margaret Cummins, the matron, initiated on her arrival.

*Present list of apparatus*: Six Bristowe coils, four galvanic switchboards with metronome interrupters, two galvanic and faradic switchboards with metronome interrupters; one switchboard for sinusoidal faradisation; one Schnee leg and arm bath; condenser, galvanic, and faradic apparatus for muscle and nerve testing; high-frequency apparatus; diathermy apparatus; apparatus for ultra-violet ray treatment.

There are also one electric-light bath for whole body; electric-light baths for local treatment; vibratory massage instrument; baths for immersing limbs in hot water; one triple whirlpool leg-bath with compressed air; one triple arm-bath to match; lastly a Tait Mackenzie and Barclay's mechano-therapeutic apparatus. The gymnastic apparatus from the old massage teaching department is also available.

Although one regrets the loss of such a palatial recreation-room, for the sailors and soldiers especially, it was obviously adapted for the purpose, and involved no structural alterations; so it had to be put to its present use.

